# 

**Published:** 1888-05-26

**Authors:** 


### THE HOSPITAL

A WEEKLY INSTITUTIONAL JOURNAL OF
### Science, Medicine, Nursing, and Philanthropy.

### CONTENTS.

**Legislative Social Reforms** - - - - - - 117

**American Jottings** - - - - - - 118

**Labourers' Wages and Labourers' Homes.** By the Right Hon. EARL GREY, K.G., G C.M.G. - - - 119

**Physical Improvement a id Technical Training** - 120

**The Doctor's Pulpit** - - - - - - 122

**Free Rest for Hospital Nurses** - - - - 123

**Notes and News** - - - - - - 124

**Annotations.**—Asylum Attendants.—Faults on both Sides.—The Public Demands an Inquiry - - - 127

**Everybody's Page** - - - - - - 126

**An Ishmaelite.** By LESLIE KEITH Author of "The Chilcotes," "East and West," etc - - - 128

**Turning Over New Leaves.**—Irish Novel.—Medical Nursing.—A Guide to Massage.—Public Hea'th.—Prosperity or Pauperism - - - - - 129

**Patients' Contributions to Hospitals.** By Mr. W. BURDETT-COUTTS, M.P. - - - - - 131

**Amusements with Profit for all Ages.**—For Men and Women.—Children's Corner.—Puzzles, etc. - - 132

**EXTRA SUPPLEMENT.—THE NURSING MIRROR.**

En Passant.—Metropolitan Nursing Association —Nurses on Strike.—Lady Doctors in Germany.—The Queen's Nursing Fund —Ealing Cottage Hospital.—The Zenana Missi.n.—Nursing in America.—Removed to the Hospital - - xxv
Original Articles.—Country Nurses - - - - - xxvi
A Nurse's Note book—Everybody's Op nion - - - xxvii
Lectures.—Appointments.—Vacancies.—Notes and Que.ies —Ex-change and Mart - - - - - - - xxviii

